# The role of the reef flat in coral reef trophodynamics: Past, present, and future

**DOI:** 10.1002/ece3.3967

**Published:** 2018-03-26

**Authors:** David R. Bellwood, Sterling B. Tebbett, Orpha Bellwood, Michalis Mihalitsis, Renato A. Morais, Robert P. Streit, Christopher J. Fulton

**Affiliations:** ^1^ ARC Centre of Excellence for Coral Reef Studies James Cook University Townsville Queensland Australia; ^2^ College of Science and Engineering James Cook University Townsville Queensland Australia; ^3^ Research School of Biology The Australian National University Canberra ACT Australia

**Keywords:** evolution, herbivory, hydrodynamics, productivity, reef fish, sediment

## Abstract

The reef flat is one of the largest and most distinctive habitats on coral reefs, yet its role in reef trophodynamics is poorly understood. Evolutionary evidence suggests that reef flat colonization by grazing fishes was a major innovation that permitted the exploitation of new space and trophic resources. However, the reef flat is hydrodynamically challenging, subject to high predation risks and covered with sediments that inhibit feeding by grazers. To explore these opposing influences, we examine the Great Barrier Reef (GBR) as a model system. We focus on grazing herbivores that directly access algal primary productivity in the epilithic algal matrix (EAM). By assessing abundance, biomass, and potential fish productivity, we explore the potential of the reef flat to support key ecosystem processes and its ability to maintain fisheries yields. On the GBR, the reef flat is, by far, the most important habitat for turf‐grazing fishes, supporting an estimated 79% of individuals and 58% of the total biomass of grazing surgeonfishes, parrotfishes, and rabbitfishes. Approximately 59% of all (reef‐wide) turf algal productivity is removed by reef flat grazers. The flat also supports approximately 75% of all grazer biomass growth. Our results highlight the evolutionary and ecological benefits of occupying shallow‐water habitats (permitting a ninefold population increase). The acquisition of key locomotor and feeding traits has enabled fishes to access the trophic benefits of the reef flat, outweighing the costs imposed by water movement, predation, and sediments. Benthic assemblages on reefs in the future may increasingly resemble those seen on reef flats today, with low coral cover, limited topographic complexity, and extensive EAM. Reef flat grazing fishes may therefore play an increasingly important role in key ecosystem processes and in sustaining future fisheries yields.

## 1 INTRODUCTION

Most Indo‐Pacific coral reefs have four distinct reef zones: the reef slope, crest, flat, and back. These zones are well characterized in terms of their respective structural features (e.g., Done, 1983; Hopley, Smithers, & Parnell, 2007), community composition (e.g., Cheal, Emslie, Miller, & Sweatman, 2012; Russ, 1984; Wismer, Hoey, & Bellwood, 2009), and hydrodynamic properties (e.g., Fulton & Bellwood, 2005; Gove et al., 2015). Indeed, so strong are these zonation patterns that there is greater dissimilarity between reef zones 10 m apart than between assemblages in similar zones on reefs separated by thousands of kilometers (Connolly, Hughes, Bellwood, & Karlson, 2005). We may thus assume that this distinct zonation is a fundamental attribute of reefs that has been in place since the earliest origins of modern scleractinian coral groups in the early Paleogene (65–23 million years ago; Bellwood, Goatley, & Bellwood, 2017). However, recent evidence suggests that the expansive reef flat may be a relatively recent feature of modern scleractinian‐dominated reefs arising about 8 million years ago (Bellwood, Goatley, Brandl, & Bellwood, 2014a; Renema et al., 2015; Santodomingo, Renema, & Johnson, 2016). Indeed, it appears that the colonization of shallow waters by grazing fishes may have triggered both the formation of the reef flat and a major shift in coral reef trophodynamics (reviewed in Bellwood et al., 2017). However, the ecological role of the reef flat in modern coral reef trophodynamics is poorly understood, with several lines of evidence suggesting that this wave‐swept coral reef zone is of limited ecological value.

**Figure 1:**
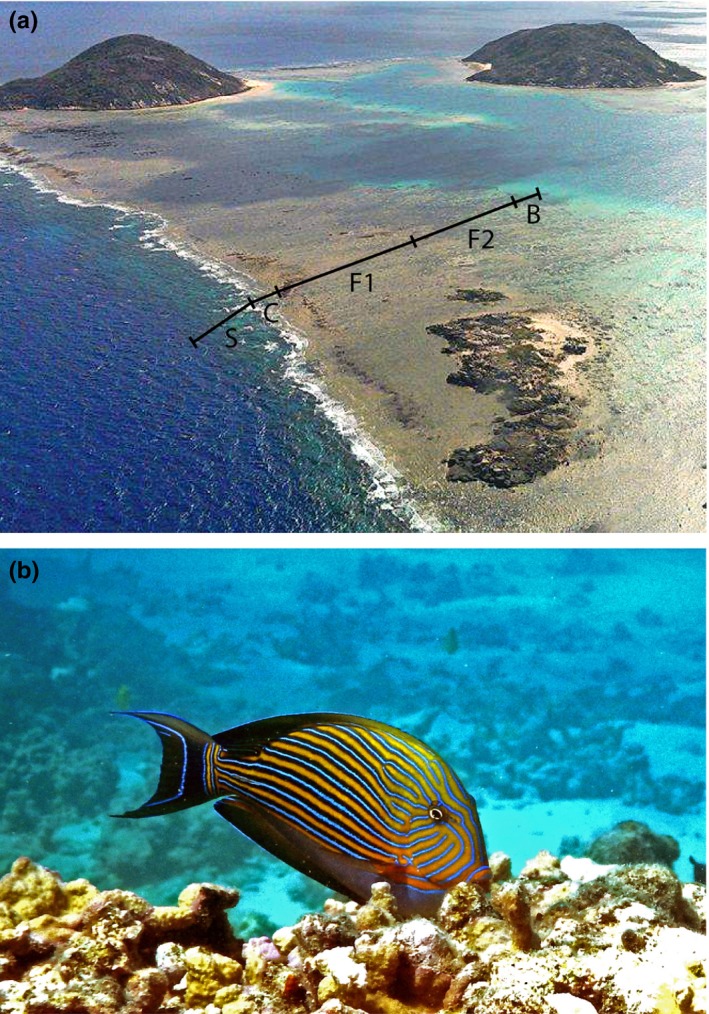
(a) Coral reef at Lizard Island on the Great Barrier Reef showing the substantial area occupied by the reef flat (C—crest; F1—mid and outer flat; F2—entire flat; B—back reef). (b) The herbivorous surgeonfish *Acanthurus lineatus*, grazing on the epilithic algal matrix (EAM)

A coral reef flat may be defined as an extensive shallow area of the reef, bounded seaward by the reef crest (the crest being the transitional area between the flat and the upper reef slope), and leeward by the back reef (cf. Done, 1983; Figure 1a). The reef flat is usually the shallowest submerged portion of a coral reef. Commonly 10s to 100s of meters wide, the flat is characterized by strong unidirectional water flow as waves break on the crest or seaward (outer) margin of the flat before passing over the rest of the flat, where water movement slowly attenuates due to friction (Kench & Brander, 2006). The reef flat benthos is often covered by relatively thick sediment‐laden algal turfs (Purcell & Bellwood, 2001), but it can support a variable density of corals, coralline algae, or macroalgae, depending on the geographic location and tidal regime (Done, 1983; Wismer et al., 2009). The reef flat also lies in the zone of highest solar irradiance, supporting significant algal growth, calcification, and primary productivity (Barnes & Devereux, 1984; Hatcher, 1988; Steneck, 1997; Wiebe, Johannes, & Webb, 1975).

From an evolutionary perspective, in terms of the structure of coral reefs, there is little evidence of significant reef flat formation by scleractinian‐dominated coral reefs prior to the Miocene (reviewed in Bellwood et al., 2017), and most large modern high‐relief reef structures are reported in the later Miocene after 8 Ma (e.g., Mihaljevi, Renema, Welsh, & Pandolfi, 2014; Renema et al., 2015; Santodomingo et al., 2016). All modern reef fish genera and smaller lineages were present long before this time (Bellwood et al., 2017). In terms of the EAM‐feeding fishes, these genera and lineages appear to have moved into shallow waters, with associated changes in body and fin morphologies (Bellwood et al., 2014a). This move is likely to have changed fish grazing patterns, leading to more intense grazing in shallow productive areas. It has been hypothesized that the presence of high‐intensity shallow‐water grazing by fishes would change the nature of coral–algal interactions, facilitating the expansion of corals (for the first time) into shallow waters (Bellwood et al., 2017). Once corals are able to dominate in shallow waters and form a consolidated wave‐resistant reef crest, subsequent infilling and progradation of the reef slope and crest would result in the formation of a reef flat. Thus, EAM‐feeding fishes may have permitted or facilitated the initial formation of the reef flat as a distinct reef habitat on scleractinian‐dominated reefs (Bellwood et al., 2014a, 2017; Brandl, Robbins, & Bellwood, 2015). The development of the reef flat as an expansive habitat of significant primary productivity, and its occupation by large numbers of grazing fishes, had the potential to revolutionize coral reef trophodynamics.

However, the potential benefits of reef flat colonization for fishes are not that obvious. Most evidence to date suggests that the reef crest, rather than the flat, is the preferred location for grazing fishes. The crest has the highest diversity of fishes (Russ, 1984; Wismer et al., 2009), extensive territoriality (with fishes protecting preferred feeding locations) (Choat & Bellwood, 1985), the highest rates of primary productivity (Klumpp & McKinnon, 1989; Russ, 2003; Steneck, 1997), and the highest detrital quality (Crossman, Choat, Clements, Hardy, & McConochie, 2001; Purcell & Bellwood, 2001).

In contrast to these beneficial characteristics of the reef crest, the conditions on the reef flat appear to limit the locomotion, feeding, and survival of fishes, with evidence of intolerably high‐sediment loads, strong water currents, and high predation risks. Studies of sediments in turf algae (Goatley & Bellwood, 2012; Purcell & Bellwood, 2001) suggest that the reef flat may have such high‐sediment loads that grazing by fishes is suppressed (Bellwood & Fulton, 2008). It has also been postulated that dynamic wave‐swept water movements may restrict reef flat access to species that either hide in flow refuges (Johansen, Bellwood, & Fulton, 2008; Johansen, Fulton, & Bellwood, 2007) or use specialized fins (Bejarano et al., 2017; Bellwood & Wainwright, 2001; Fulton, Wainwright, Hoey, & Bellwood, 2017). High‐aspect‐ratio pectoral fins and the capacity to use adaptive shifts in swimming behavior (e.g., increased use of stabilizing median fins, changing body posture to minimize flow‐induced drag) appear to be particularly important for fishes to move with efficiency and stability in these rapidly changing and often extreme flow environments (Fulton, Johansen, & Steffensen, 2013; Heatwole & Fulton, 2013; Webb, Cotel, & Meadows, 2010). Finally, although direct evidence of predation on adult fishes is limited, there appears to be a high risk of predation in this zone, with several studies identifying high predation rates as a possible explanation for the low fish abundance of some fish groups on the reef flat (e.g., Fox & Bellwood, 2007; Hay, 1981; Khan, Welsh, & Bellwood, 2016).

There are therefore two conflicting views of the reef flat as a fish habitat: (1) an evolutionary breakthrough into an area of high primary productivity, or (2) an undesirable high‐sediment, high‐energy, high‐risk location, which is detrimental to fish populations. The goal of this study, therefore, is to reconcile these two conflicting characterizations of the reef flat by quantifying the relative importance of the reef flat in modern coral reef ecosystems. By focusing on grazing reef fishes, we specifically examine the reef flat's role in reef trophodynamics within the context of the previously posited challenging environmental characteristics.

## 2 MATERIALS AND METHODS

To quantify fish abundance in a relatively undisturbed reef system, we surveyed two cross‐shelf transects on the Great Barrier Reef (GBR) in the northern (2004) and central (2005) sectors, before recent major cyclones (Khan, Goatley, Brandl, Tebbett, & Bellwood, 2017) or bleaching events (Hughes et al., 2017a). In each sector, fishes were surveyed on two reefs in each shelf location, with four transects per habitat per reef. This level of replication clearly describes major patterns across and within reefs (see Wismer et al. (2009) for details of reef locations, and Bellwood and Wainwright (2001) on broad cross‐shelf patterns). Fishes were censused in the four major reef zones—slope (at 12 m depth), crest (at 2–5 m), flat (2–5 m; approximately 20 m in from the crest), and back reef (at 2–5 m along the leeward reef margin)—using 10‐min timed belt transects equating to approximately 117 m (calibrated following Bellwood and Wainwright (2001)). These timed transects were specifically developed to get accurate estimates of larger reef fish species that exhibit strong diver‐negative effects, such as parrotfishes (Dickens, Goatley, Tanner, & Bellwood, 2011; Welsh & Bellwood, 2012). Dickens et al. (2011) reported a 70% underestimation in parrotfish counts when using traditional belt transects involving multiple passes to lay transect tapes before fish counting.

Our focus here is on those species that predominantly graze the epilithic algal matrix (EAM) on hard substrata. By excluding species that feed on other benthic resources, we focus on the direct link between algal (EAM) primary productivity and fish biomass. We, therefore, only included those species in the Acanthuridae (7 species) (Figure 1b), Labridae (Tribe Scarini; i.e., parrotfishes) (18), and Siganidae (4) that graze the EAM (grazing is taken as a general term to include croppers and scrapers) (Table S1). The species are identified as predominantly EAM feeders (following Brandl et al., 2015; Choat, Clements, & Robbins, 2002; Green & Bellwood, 2009; Hoey, Brandl, & Bellwood, 2013; Kelly et al., 2016; Russ, 1984). We excluded specialist detritivores (Tebbett, Goatley, & Bellwood, 2017a), acanthurids which feed over mixed or soft substrata, that is, “sediment suckers” (sensu Russ, 1984), excavating parrotfishes that may target corals (Bellwood, Hoey, & Choat, 2003) or endolithic material (Clements, German, Piché, Tribollet, & Choat, 2017), macroalgal browsers (Streit, Hoey, & Bellwood, 2015), and planktivores, as these species are not necessarily feeding on EAM productivity per se. The feeding locations of macroalgal browsers are hard to determine and may include interreefal habitats (Lim, Wilson, Holmes, Noble, & Fulton, 2016; Marshell, Mills, Rhodes, & McIlwain, 2011; Pillans et al., 2017). Our values are therefore a conservative estimate of the total productivity from our focal habitats.

Fishes were counted by two divers on SCUBA, the first diver counted fish >10 cm total length (TL) in a 5‐m‐wide transect and the other fish <10 cm TL in a 1‐m‐wide transect. Both divers placed fishes into TL size categories (5 cm for fishes >10 cm and 2.5 cm for fishes <10 cm). These transects were combined into a single area‐standardized abundance metric (100 m^−2^). All censuses were conducted at mid to high tide, at least 1 m above chart datum. This ensured that counts were undertaken when the shallowest areas were all available for feeding (the reef flat and crest may become unavailable at low tides (<0.3 m) with a lack of water forcing fish to feed elsewhere). It is estimated that the flat would be covered by less than 0.3 m of water for approximately 0.4% of daylight hours (Text S1). Details of the methods are given in Bellwood and Wainwright (2001) and Wismer et al. (2009), with a full description of each site in Wismer et al. (2009).

To calculate biomass, we used Bayesian length‐weight regression parameters for each species from Fishbase (Froese & Pauly, 2017; Froese, Thorson, & Reyes, 2014). Bayesian length‐weight coefficients have the advantage of combining information from independent studies and, as such, are less sensitive to measurement errors. We used the Bayesian length‐weight regression parameters to calculate grazer biomass (grams [g] 100 m^−2^). Daily biomass growth of grazing fishes (g 100 m^−2^ day^−1^) was estimated by applying the von Bertalanffy growth model (VBGM) to censused fishes following the procedure described by Depczynski, Fulton, Marnane, and Bellwood (2007). Published VBGM coefficients were derived from growth studies carried out on the GBR (Choat & Robertson, 2002; Gust, Choat, & Ackerman, 2002). In addition, the algal turf productivity consumption by grazers (g carbon [C] day^−1^) was calculated based on the equation γ = 27.78χ + 2.79 (from figure 2 in Russ, 2003) where γ is the biomass of grazing fishes (grams of wet weight m^−2^) and χ is the algal yield to grazing fishes (gC/day m^2^). It should be noted that this equation does not address size‐specific consumption or metabolic rates of grazing fishes, just an overall relationship for grazing fish assemblages (surgeonfishes, rabbitfishes, and parrotfishes).

Grazer abundance, biomass, algal productivity consumed, and grazing fish biomass growth were also calculated after standardizing for the relative area of each reef zone (i.e., value 100 m^−2^ × percentage of total relevant reef area occupied by specific reef zones: slope, crest, flat, back.). The effects of tides on algal consumption was examined by assuming cessation of feeding by reef flat individuals at 0.3 m of water and relocation of flat individuals equally to the slope, crest, and back. Displaced fishes from the flat often school, without feeding, in the reef crest area. However, to examine the potential effect of tides, we assumed that all fishes feed at the same rates but in the new locations.

The relative areas of the reef zones are based on satellite photographs (Google Earth) of ten haphazardly selected mid‐ and outer‐shelf reefs from the central/northern GBR. Reefs were selected where satellite resolution and reef configurations permitted clear habitat delineation (where possible these reefs included the reefs where fish were censused). Areas of nonhorizontal reef zones (i.e., slopes) were calculated using trigonometry (based on nautical charts, a depth of 20 m was used for reef slopes and 6 m for back reefs). In the images, the reef crest was identified as the pale outer margin on the windward edge of each reef. The crest was separated from the slope by a distinct darkening in color as the water deepened, and in a leeward direction from the flat as the substratum darkened in color. Where waves were present in the photo the initiation of the wave break was also used to help identify the reef crest (following description in Fulton & Bellwood, 2005). This method may overestimate the size of the crest; however, this is likely to make our estimates for the reef flat more conservative. The back reef was identified as the continuous outer margin of the leeward edge of the reef, which stretched from the rear of the flat (lighter substratum) into deeper water (mirroring the seaward crest).

Our focus was on mid‐ and outer‐shelf reefs because inner‐shelf reef habitats have less clearly delineated zones (Bellwood & Wainwright, 2001). We follow most previous studies (Done, 1983; Wismer et al., 2009) in regarding the crest and flat as separate habitats. The area of the reef flat was measured twice: (1) conservatively, covering only the mid and outer flat (cf. Fox & Bellwood, 2007) (where corals are found on the seaward edge and continue to provide some cover on the flat) and (2) incorporating the outer/mid flat, as above, but also including the leeward section where the reef matrix is interspersed with sand areas (Fulton & Bellwood, 2005; see Figure 1a; Table S2 for full details of reefs used and widths of habitats). Only the conservative estimates are used for analysis herein (see Table S2 for differences between conservative and nonconservative estimates).

Differences among reef zones in the abundance, biomass, algal productivity consumed, and biomass growth of grazing fishes (per unit area [100 m^2^] and total values for each reef habitat) were examined using generalized linear mixed‐effects models (GLMMs) with a negative binomial distribution (abundance data) or gamma distribution (biomass, biomass growth data, and productivity consumed) with a log link function (all models). In all cases, shelf position (mid and outer) and reef habitat (back, flat, crest, and slope) were treated as fixed effects, while reef was treated as a random factor nested within shelf position. Models were simplified to the most parsimonious solution using the corrected Akaike information criterion (AICc [Table S3]). Statistical modeling was performed in R (R Core Team 2014) using the *lme4* (Bates, Maechler, Bolker, & Walker, 2015) and *AICcmodavg* (Mazerolle, 2015) packages.

## 3 RESULTS

**Figure 2:**
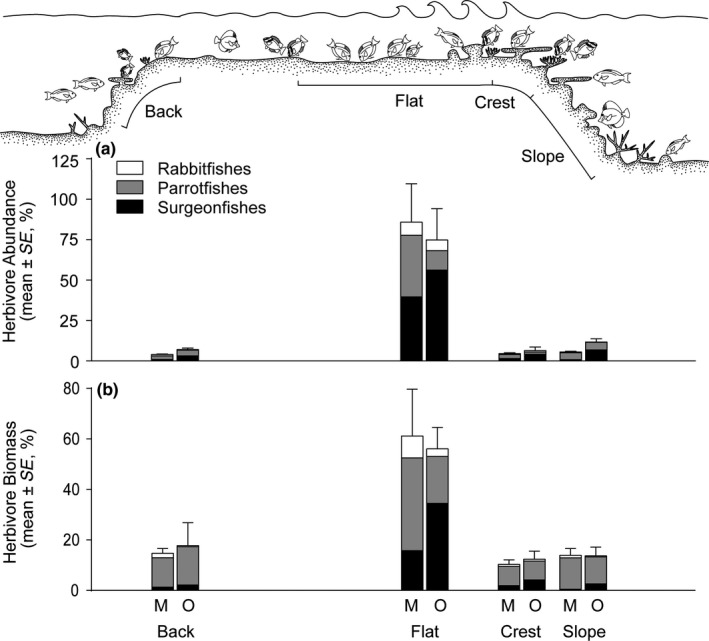
The distribution of grazing herbivorous fish (a) abundance and (b) biomass, in four reef habitats across two shelf positions (mid [M] and outer [O]) on the Great Barrier Reef. Values expressed as a percentage of total grazer abundance/biomass in each habitat in each shelf position (i.e., abundance m^−2^ × area of habitat, as a percentage of total abundance over all areas in each shelf position), see supporting information for proportions for each family separately and observed densities (individuals or biomass 100 m^−2^, Figures S1a,b, S2a,c,d)

The high wave energy reef flat and crest zones supported 2.4 to 4.8 times more individuals and 1.5 to 1.7 times more grazing fish biomass per unit area than either of the two low energy zones, that is, the back reef and slope (Figure S1c,d; Table S4). However, if the relative area of the various habitats is taken into consideration, the overwhelming contribution of the reef flat to fish populations and ecosystem processes is revealed (Figure 2). On average, the reef flat covers 1.2 times the area of all other habitats combined. After accounting for fish densities and the area of the reef flat, the reef flat alone supports up to 92.6%, 80.8%, and 82.8% of all grazing surgeonfishes, parrotfishes, and rabbitfishes, respectively; estimates vary with shelf location (Figure S2a,c,e). These values refer to all fishes between 20 m on the slope to 6 m deep on the back reef. In biomass, this equates to up to 80.8% of all grazing surgeonfish biomass, 53.7% of parrotfish and 71.4% of rabbitfish biomass (Figure S2b,d,f). For all grazers combined, the reef flat supports up to 85.9% of individuals and 61.1% of total fish biomass (with an average of 78.8% and 57.8%, respectively) (Figure 2b). If the crest and flat values are combined, these shallow high‐energy locations can support up to 90.4% of all individuals and 71.4% of total grazing fish biomass. Grazing fish abundance and biomass both exhibit a strong, positive, correlation with wave‐induced water motion (net current velocity) (Text S2, Table S5, Figure S3). The GLMMs indicated that the reef flat (standardized for area) has a significantly higher abundance and biomass of herbivorous grazing fishes compared to all other habitats (GLMM; *p *<* *.001 in all cases: Table S4).

**Figure 3:**
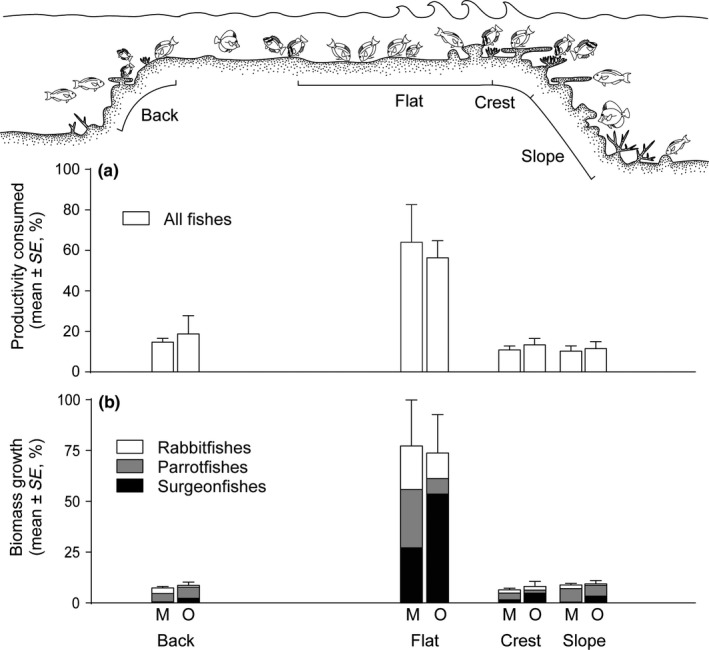
(a) The percentage of algal turf productivity (gC/day) consumed by grazing herbivorous fishes and (b) the percentage of grazing herbivorous fish biomass growth (g/day) in four reef habitats across two shelf positions (mid [M] and outer [O]) on the Great Barrier Reef. Values expressed as a percentage of total productivity consumed/biomass growth in each habitat in each shelf position (i.e., productivity consumed m^−2^ × area of habitat, as percentage of total abundance over all areas in each shelf position). See supporting information for values (per 100 m^2^, Figures S1c,d, S2b,d,f)

The importance of the reef flat for coral reef ecosystems is also clearly seen in terms of the dynamic processes operating in these shallow high‐energy locations, that is, algal productivity consumption and daily biomass growth of grazing fishes. When standardized by habitat area, the reef flat alone accounts for up to 64.1% of all algal productivity consumed (gC/day) by grazing fishes (Figure 3a). If the other wave‐swept zone (i.e., the reef crest) is included, this estimate rises to 75%. Fish biomass growth is equally remarkable, with up to 77.3% of all grazer biomass growth occurring on the reef flat (Figure 3b). If the crest and flat values are combined, the two shallow habitats together support up to 83.8% of the total (g/day) increases in grazer biomass. The GLMMs indicated that the reef flat (standardized for area) has significantly higher (1) consumption of algal productivity by grazing fishes and (2) biomass growth of grazers, compared to all other habitats (GLMM; *p *<* *.001 in all cases: Table S4). These results are robust to the effects of tides. If grazing is assumed to cease at 0.3 m water depth the daily grazing on the flat fell from 58.89% to 58.66%, while the others rose to just 12.56% on the crest (from 12.48%), 11.35% on the slope (from 11.27%) and 17.44% on the back (from 17.36%).

## 4 DISCUSSION

The reef flat is by far the most important area on GBR coral reefs for EAM‐grazing fishes, supporting approximately 79% of individuals, 58% of fish biomass, 59% of turf algal productivity consumption, and 75% of fish biomass growth. The wave‐swept reef flat and/or crest also supports the highest m^2^ densities of grazing herbivorous fishes. These findings are in broad agreement with previous studies that reported relatively high abundances of nominally herbivorous fishes in the shallowest areas of the reef, usually the crest, on both GBR (Russ, 1984; Wismer et al., 2009) and Caribbean reefs (Hay, 1981; Steneck, 1983, 1988). However, from an ecosystem perspective, it is the size of the contribution of the reef flat to both fish populations and grazer trophodynamics that is most notable. While a high abundance of reef flat grazing fishes is apparent in terms of densities (ind. 100 m^2^), it is only when total available habitat area is taken into consideration that the remarkable role of the reef flat in coral reef trophodynamics becomes apparent. The reef flat zone alone supports an estimated 79% of individuals and 75% of biomass growth in this important group of GBR coral reef algal consumers.

The reef flat is the largest coral reef habitat by area (excluding the sediment apron and off‐reef mesophotic zones). It supports the highest grazing fish biomass and, presumably, the highest total productivity to maintain this grazing activity (Hatcher, 1981; Russ, 2003; Steneck, 1997; Wiebe et al., 1975). High EAM‐based productivity in this zone was highlighted by (Bonaldo & Bellwood, 2011), who showed total regrowth of algae after grazing in just 3 days. Together, the crest and flat support up to 72% of all turf algal‐based carbon consumed by EAM‐grazing fishes (Figure 3a). Fishes leave the flat to sleep and spawn (Fox, Bellwood, & Jennions, 2015; Welsh & Bellwood, 2012), transferring nutrients as excreta, gametes, or somatic tissue to contribute to food chains elsewhere on the reef, including detrital food chains in back reef or lagoonal environments (Crossman et al., 2001; Vermeij et al., 2013). The reef flat therefore acts as a major pathway for primary productivity that supports higher‐level trophic networks, including those in deeper parts of the coral reef.

### 4.1 EAM‐grazing fishes as an indicator of reef trophodynamics

This high concentration of EAM‐grazing fishes on coral reef flats agrees well with earlier evidence. Steneck (1997) noted that these shallow areas are the most highly productive locations on the reef and, as noted by Russ (2003), fish biomass seems to match algal productivity. The spatial patterns of EAM‐grazing fishes recorded here also match those seen for herbivores in general (Bejarano et al., 2017; Wismer et al., 2009). However, because these earlier studies included non‐EAM‐feeding species (including browsers, and particulate feeders), the trophic linkages were less clear. By focusing on EAM‐grazing fishes only, we are able to focus on the direct link between reef‐based primary productivity and fish biomass and hence identify the outstanding importance of this extensive shallow habitat in reef trophodynamics.

This study only considers primary productivity from algal turfs within the EAM. In some areas, the reef flat may also support a significant biomass of macroalgae (Fox & Bellwood, 2007; Kobryn, Wouters, Beckley, & Heege, 2013; Wismer et al., 2009). If the contribution of these algae is added to the turfs, the overall contribution of this area to total reef productivity is likely to be significantly greater than in our estimates based on EAM alone. Of all reef habitats, the reef flat is most frequently the area with the greatest coverage of macroalgae (Fox & Bellwood, 2007; Hay, 1981; Wismer et al., 2009). However, with macroalgae there are additional problems when calculating yields to herbivores because of: (1) the need to separate material lost due to breakage from that consumed by herbivores (Fulton et al., 2014; Lim et al., 2016) and (2) the potential disconnect between the presence of browsers and their feeding activity.

When considering reef trophodynamics it is important to note that fish presence may not always equate to feeding activity. Studies have repeatedly shown that herbivorous fishes are often not seen in underwater visual census transects (Dickens et al., 2011), and are only detected using video techniques (Bellwood, Hughes, & Hoey, 2006; Bennett & Bellwood, 2011; Cvitanovic & Bellwood, 2009; Vergés, Bennett, & Bellwood, 2012). However, this underestimation of herbivory is primarily seen in macroalgal browsers. Evidence to date suggests that EAM grazers, which were the focus of our analysis, are relatively apparent on visual censuses (diver effects notwithstanding), and that they are likely to exert a grazing pressure that is broadly in proportion to their apparent densities (Fox & Bellwood, 2008). Our focus on EAM‐grazing fishes, therefore, allows direct inferences from observed fish presence to trophic links between primary productivity and fish biomass.

Our results suggest that the reef flat is a good habitat for EAM‐grazing fishes. However, this raises questions over the reported drawbacks of the reef flat zone: high‐sediment loads, hydrodynamic challenges to locomotion, and enhanced predation risks. This paradoxical situation may be tied to the trophodynamics, feeding modes, and evolutionary history of the fishes that exploit this zone.

### 4.2 The reef flat: ecological challenges and evolutionary solutions

It has been posited that colonization of shallow high‐energy locations, and the reef flat in particular, during the Miocene was advantageous for herbivorous reef fishes (Bellwood et al., 2014a, 2017; Brandl & Bellwood, 2014). However, the extent of any advantage has not been quantified. Our data strongly suggest that the occupation of shallow waters, and the reef flat in particular, is beneficial. Indeed, up to 93% of grazing surgeonfishes, 83% of rabbitfishes and 81% of parrotfishes were found to live on the reef flat. Overall, this equates to approximately 79% of individuals and 58% of the total grazer biomass. This capacity to utilize shallow‐water resources potentially allowed fishes to increase their populations by over 900% (i.e., the mid‐shelf flat and crest contain 90.4% of individuals vs. 9.6% on the slope and back; Text S3). The benefits of reef flat occupation may include: higher fish growth rates as a result of access to highly productive fast growing algae (Choat & Axe, 1996; Depczynski et al., 2007) or high‐quality cyanobacteria (Clements et al., 2017); fewer secondary metabolites (Hay & Fenical, 1988); year‐round productivity (Wilson, Bellwood, Choat, & Furnas, 2003); less competition from grazing invertebrates (Steneck, Bellwood, & Hay, 2017) and higher quality detritus in the EAM (Crossman et al., 2001; Purcell & Bellwood, 2001). These lines of evidence all suggest that reef flat occupation is advantageous.

Nevertheless, there are also a number of disadvantages. In some areas, for example, tidal cycles can have a significant influence on both fish behavior and reef access during extreme tides (Harborne, 2013). In our study areas, the reef flats are approximately at chart datum. They may therefore be unavailable for grazing during part of the tidal cycle. Although grazing fishes will often continue to feed until their backs or tails are exposed to the air it is plausible that some fishes may cease to feed as water levels fall. If feeding ceases at 30 cm water depth (an arbitrary figure to explore the magnitude of the effect) it is estimated that the flat would be unavailable for feeding for approximately 0.4% of daylight hours (Text S1), which had a negligible effect on the ecosystem processes described herein. It is high‐sediment loads, strong water movement and high predation risks that appear to be the primary influences on herbivorous fish distributions (Bejarano et al., 2017; Bellwood & Fulton, 2008; Fox & Bellwood, 2007; Hay, 1981).

Sediments are known to deter herbivory (Bellwood & Fulton, 2008; Clausing et al., 2014; Tebbett, Goatley, & Bellwood, 2017b) and, of all locations on a reef, the flat has the highest sediment loads (Purcell & Bellwood, 2001). The solution to this paradox, that is, why fishes feed in apparently undesirable high‐sediment locations, may lie in the distinct feeding mechanisms of EAM‐grazing fishes. Functional analyses of feeding in nominally herbivorous fishes suggest that some fishes are not as constrained by sediments as others. Specialist species that eat particulates (e.g., *Ctenochaetus striatus*) and those that eat the whole EAM (i.e., parrotfishes) appear to be most deterred by sediments (Bellwood & Fulton, 2008; Gordon, Goatley, & Bellwood, 2016; Tebbett, Goatley, & Bellwood, 2017c; Tebbett et al., 2017b). By contrast, grazing *Acanthurus* eat algae above the sediment layer and are thus not as constrained by sediments (Tebbett et al., 2017a, 2017b). The answer to the sediment paradox, therefore, may be that for fish species that are able to avoid ingesting sediments, the reef flat is a highly advantageous and productive feeding location.

In this respect, it is noteworthy that tooth morphologies associated with algal grazing were present in surgeonfishes and rabbitfishes in the Eocene, while the specialist dentition associated with particulate or whole‐EAM‐feeding arose much later in the Miocene (Bellwood, Hoey, Bellwood, & Goatley, 2014b; Bellwood et al., 2017). It is therefore likely that grazing fishes possessed dentition that enabled them to avoid sediments during feeding, long before reef flats arose as a major reef habitat in the late Miocene c. 8 Ma (Bellwood et al., 2017; Renema et al., 2015; Santodomingo et al., 2016).

This paper specifically considers the role of sediments on relatively wave‐exposed reef flats (mid and outer‐shelf GBR reefs). However, there are two distinct reef types on the GBR: exposed reefs where sediments are largely biogenic and coastal reefs where inorganic (siliceous) terrigenous inputs can have a major influence (Tebbett, Goatley, & Bellwood, 2017d). In these areas, fine inorganic sediments appear to suppress both herbivory and detritivory (Cheal, Emslie, MacNeal, Miller, & Sweatman, 2013; Goatley, Bonaldo, Fox, & Bellwood, 2016; Tebbett et al., 2017c) and, as a consequence, the capacity of coastal reefs to support reef flat and fish populations may be limited. There is increasing evidence that coastal reefs have distinct herbivorous fish assemblages (Cheal et al., 2012; Hoey et al., 2013; Johansson, van de Leemput, Depczynski, Hoey, & Bellwood, 2013; Wismer et al., 2009) and that this may in part be driven by the ability of these fishes to tolerate fine sediments (Gordon et al., 2016). With reefs transforming due to anthropogenic pressures (Hughes et al., 2017b), the role of sediments on inshore reefs is likely to become an increasingly important concern.

The evolutionary and ecological evidence both suggest that strong water movement may restrict occupation of the reef flat and that the benefits of living there may be increased by modifications to the locomotor system. In extant fishes, it appears that one of the key adaptations that underpinned effective colonization of the reef flat was labriform locomotion using high‐aspect‐ratio pectoral fins (Bellwood & Wainwright, 2001; Fulton, Bellwood, & Wainwright, 2001; Fulton et al., 2017). Fossil evidence indicates that early labrids all had low‐aspect‐ratio fins (Bannikov & Bellwood, 2015), while phylogenetic evidence indicates that fish groups with high‐aspect‐ratio fins probably arose in the Miocene, in both the Labridae and Acanthuridae (Bellwood et al., 2017; Cowman, Bellwood, & van Herwerden, 2009; Sorenson, Santini, Carnevale, & Alfaro, 2013). Today, the most successful fishes on the reef flat are surgeonfishes and the wrasse genus *Thalassoma*. These fishes possess some of the highest pectoral fin aspect ratios seen among coral reef fishes (Fulton & Bellwood, 2005; Fulton et al., 2017). Thus the fishes that predominate on the reef flat today have an evolutionary history that points to the acquisition of efficient propulsion in high‐energy locations (high‐aspect‐ratio fins) as a key feature that facilitated reef flat colonization.

Evolutionary and ecological evidence also suggests that the reef flat can be, and probably always was, a dangerous place (Bellwood et al., 2014a, 2017; Fox & Bellwood, 2007; Hay, 1981; Lewis & Wainwright, 1985). Mortality in reef fishes is particularly high in young fishes when they are vulnerable to numerous predators (Goatley & Bellwood, 2016; Goatley, Gonzálex‐Cabello, & Bellwood, 2017). Mortality in large reef fishes has generally been assumed to be much lower, with the period of greatest mortality being during the night or crepuscular periods. Recently, however, Khan et al. (2016) demonstrated that predation on adult herbivorous reef fishes is not just by nocturnal or crepuscular feeders such as sharks and moray eels. Adult shallow‐water herbivorous fishes are most likely to be eaten in the day by shallow‐water high‐speed predators. Of these predators, jacks such as *Caranx ignobilis* are leading contenders, with reports of large *C. ignobilis* patrolling shallow reef areas during the day (Khan et al., 2016). It is interesting to note that in response to the colonization of the reef flat some time during the Miocene, the main morphological modifications in surgeonfishes (eye position and fin morphology) both appear to be related to predation avoidance (Bellwood et al., 2014a). Presumably, the trophic apparatus surgeonfishes used to graze in deeper water or less wave‐exposed locations was perfectly suitable to graze in shallow waters.

The reef flat, therefore, appears to be a location with high‐stakes tradeoffs. Predation and locomotion challenges are evolutionary and ecological problems that persist to this day. There is therefore no contradiction in the proposed advantages and disadvantages of the reef flat. It appears that the flat is indeed a high‐sediment, hydrodynamically challenging, and dangerous place. However, the trophic rewards make the reef flat worth occupying and over evolutionary time adaptations have allowed fishes to take advantage of this challenging environment.

### 4.3 Future implications for reefs and fisheries yields

Our observations have important ramifications for the future of coral reefs and the services they provide to humans, particularly fisheries yields. With increasing anthropogenic pressure, reefs are changing rapidly (Hughes et al., 2017b). The two most notable effects are the loss of reef‐building corals, a phenomenon that is rapidly escalating due to climate‐induced bleaching (Hughes et al., 2017b), and the subsequent loss of structural complexity, which has broad flow‐on effects for fish communities (Graham & Nash, 2013; Pratchett et al., 2008). The loss of corals results in the loss of critical habitats for large reef fishes (Kerry & Bellwood, 2015; Khan et al., 2017; Pratchett et al., 2008) and may impact fisheries yields (Graham et al., 2007). Reefs of the future are going to be unlike anything previously encountered by humankind (Hughes et al., 2017b), with fewer corals and less three‐dimensional complexity. Furthermore, there is evidence to suggest that algal productivity will be increased under future climate‐change scenarios (Bender, Champ, Kline, Diaz‐Pulido, & Dove, 2015; Ober, Diaz‐Pulido, & Thornber, 2016). All of these aspects (low coral cover, low complexity, and high algal productivity) are preexisting characteristics of the reef flat. In the future, it is likely that entire reef profiles will begin to resemble reef flats as we see them today.

As coral reefs lose coral cover, the relative importance of the reef flat, and species that can live in exposed locations, is likely to increase. Thus reef flats may hold the key to the future sustainability of reef fishery yields. Furthermore, the sustainability of reef flat species is likely to be enhanced by the resilience of the dominant shallow‐water algal grazers, parrotfishes, and rabbitfishes, to heavy fishing pressure (Bellwood, Hoey, & Hughes, 2012; Condy, Cinner, McClanahan, & Bellwood, 2015; Russ, Questel, Rizzari, & Alcala, 2015). While it has been known for a long time that reef fish yields from different reef habitats may vary considerably (Bellwood, 1988), our data also highlight the importance of specific reef zones (especially the shallow crest and flat). These observations suggest that effective management of the reef flat may be critical for the long‐term sustainability of coral reef fish yields under future climate‐change scenarios, when the loss of corals and increasing sea levels are likely to make the reef flat one of the most resilient habitats for sustaining reef ecosystem processes and reef fisheries yields.

In conclusion, the reef flat appears to be the single most important area for grazing fishes on coral reefs in terms of abundance, biomass, consumption of algal productivity, and grazing fish biomass production. The suggestion that a move on to the reef flat was a substantial improvement for fishes in the Miocene is supported by observations on modern reefs. Of the major challenges presented by the reef flat, food availability seems to have been relatively easily overcome, while water movement and predation appear to have been the primary constraints on reef flat access from the Miocene to this day. Yet, under future climate‐change scenarios, this reef habitat may become an increasingly important area for both coral reef ecosystems and reef fisheries yields.

## CONFLICT OF INTEREST

None declared.

## AUTHOR CONTRIBUTION

DRB, SBT, and OB conceived the ideas; SBT and RM analyzed the data; DRB, SBT, OB, MM, RM, RPS, and CJF interpreted the data and wrote the article. All authors contributed critically to the drafts, gave final approval for publication, and agree to be accountable for all aspects of the work.

## DATA ACCESSIBILITY

Data will be uploaded to the Tropical Data Hub, James Cook University.
