# Supplementary material for: The role of the reef flat in coral reef trophodynamics: Past, present, and future
Source: Ecol Evol. 2018 Mar 26;8(8):4108–19. doi: 10.1002/ece3.3967 (PMC5916286; doi:10.1002/ece3.3967)
Supplement: Supplementary file 1 [file ECE3-8-4108-s001.doc]

**Supporting Information**

**Table S1­­­** EAM grazing herbivorous fish species used in the analysis.

| **Family** | **Genus** | **Species** |
| --- | --- | --- |
| Acanthuridae | *Acanthurus* | *nigroris* |
|  |  | *lineatus* |
|  |  | *nigricans* |
|  |  | *nigrofuscus* |
|  |  | *triostegus* |
|  | *Zebrasoma* | *scopas* |
|  |  | *velifer* |
| Labridae | *Hipposcarus* | *longiceps* |
|  | *Scarus* | *altipinnis* |
|  |  | *chamaeleon* |
|  |  | *dimidiatus* |
|  |  | *festivus* |
|  |  | *flavipectoralis* |
|  |  | *forsteni* |
|  |  | *frenatus* |
|  |  | *ghobban* |
|  |  | *globiceps* |
|  |  | *niger* |
|  |  | *oviceps* |
|  |  | *psittacus* |
|  |  | *rivulatus* |
|  |  | *rubroviolaceus* |
|  |  | *schlegeli* |
|  |  | *spinus* |
|  |  | *sp.* |
| Siganidae | *Siganus* | *argenteus* |
|  |  | *corallinus* |
|  |  | *doliatus* |
|  |  | *spinus* |

**Text S1** Effects of tides.

To consider the effects of tides on the ability of herbivorous fishes to graze the reef flat we calculated the proportion of grazing time per year that access to the reef flat would be limited by water depth. To do this we used tide data for the Cairns region (16° 56´ S, 145° 47´ E) in the northern Great Barrier Reef, Australia for 2016 (Bureau of Meterology 2016). Based on a 12 hour “grazing day” (0600 to 1800; when herbivorous fishes may be grazing on the reef flat [Zemke-White, Choat & Clements 2002]) we calculated the percentage of time per year that the reef flat was covered by < 30 cm, < 50 cm and < 1 m of water. Calculations were based on the assumptions that: a) the reef flat is at chart datum, and b) the water level would be at the low tide level for approximately 1 hour either side of low tide. Assumption b was based on the ‘Rule of Twelfths’, which may be used as a rule of thumb to calculate the tide height at any time, relative to the time and height of high and low water (Marley, Salgado Kent & Erbe 2017). Following these assumptions we calculated how many hours per year the reef flat may be covered by less than 30 cm, 50 cm or 1 m of water as a percentage of total grazing hours per year.

We found that each year the reef flat was covered by < 30 cm of water for approximately 0.4% of the time, < 50 cm for 1.6% and < 1 m for 7.3%.

**References**

Marley, S.A., Salgado Kent, C.P. & Erbe, C. (2017) Occupancy of bottlenose dolphins (*Tursiops aduncus*) in relation to vessel traffic, dredging, and environmental variables within a highly urbanised estuary. *Hydrobiologia*, **792**, 243–263.

Bureau of Meterology (2016) Cairns Queensland times and heights of high and low tides. URL http://www.bom.gov.au/ntc/IDO59001/IDO59001_2016_QLD_TP006.pdf [accessed 17 July 2017]

Zemke-White, L.W., Choat, J.H. & Clements, K.D. (2002) A re-evaluation of the diel feeding hypothesis for marine herbivorous fishes. *Marine Biology*, **141**, 571–579.

**Table S2** The width of each reef habitat (in m) on the specific reefs measured. The relative difference in the area of reef habitats is based on a standardised distance of reef front (e.g. 100 m), as such, differences between habitats are driven by the width of each habitat.

|  |  | **Width of reef habitat (metres)** | | | | |
| --- | --- | --- | --- | --- | --- | --- |
| **Shelf Position** | **Reef** | **Back** | **Flat (conservative )** | **Flat (*sensu lato*)** | **Crest** | **Slope** |
|  |  |  |  |  |  |  |
| **Mid** | **Lizard** | 30.32 | 180.52 | 342.14 | 21.02 | 47.22 |
|  |  |  |  |  |  |  |
| **Mid** | **Macs** | 54.40 | 180.91 | 949.79 | 19.81 | 40.59 |
|  |  |  |  |  |  |  |
| **Mid** | **Irene Reef** | 38.23 | 131.81 | 960.33 | 21.7 | 116.18 |
|  |  |  |  |  |  |  |
| **Mid** | **Bramble Reef** | 19.52 | 176.92 | 167.74 | 21.19 | 136.63 |
|  |  |  |  |  |  |  |
| **Mid** | **Helix Reef** | 59.32 | 146.3 | 513.7 | 23.49 | 76.36 |
|  |  |  |  |  |  |  |
| **Mid** | **Davies Reef** | 11.33 | 68.3 | 169.33 | 10.33 | 32.58 |
|  |  |  |  |  |  |  |
| **Mid** | **Big Broadhurst Reef** | 21.31 | 142.19 | 380.61 | 15.17 | 67.67 |
|  |  |  |  |  |  |  |
| **Mid** | **Mid Reef** | 51.66 | 102.41 | 414.46 | 22.2 | 114.48 |
|  |  |  |  |  |  |  |
| **Mid** | **Stanley Reef** | 24.05 | 111.37 | 385.49 | 20.47 | 82.63 |
|  |  |  |  |  |  |  |
| **Mid** | **Peart Reef** | 50.0 | 81.93 | 388.81 | 14.43 | 54.2 |
|  |  |  |  |  |  |  |
| **Outer** | **Ribbon 10** | 35.24 | 197.73 | 574.28 | 20.8 | 48.63 |
|  |  |  |  |  |  |  |
| **Outer** | **Ribbon 9** | 44.07 | 213.6 | 628.79 | 21.39 | 39.57 |
|  |  |  |  |  |  |  |
| **Outer** | **Ribbon 2** | 32.87 | 291.06 | 402.03 | 22.32 | 129.75 |
|  |  |  |  |  |  |  |
| **Outer** | **Lena Reef** | 38.13 | 168.81 | 308.58 | 26.09 | 160.98 |
|  |  |  |  |  |  |  |
| **Outer** | **Anderson Reef** | 50.82 | 208.34 | 915.33 | 24.11 | 140.59 |
|  |  |  |  |  |  |  |
| **Outer** | **Agincourt Reefs** | 24.47 | 147.28 | 401.42 | 18.69 | 95.09 |
|  |  |  |  |  |  |  |
| **Outer** | **Arlington Reef** | 57.57 | 153.13 | 621.86 | 22.92 | 173.66 |
|  |  |  |  |  |  |  |
| **Outer** | **Half Moon Reef** | 40.01 | 200.49 | 444.83 | 16.71 | 73.31 |
|  |  |  |  |  |  |  |
| **Outer** | **Beacon Reef** | 40.67 | 206.62 | 1130 | 21.63 | 129.49 |
|  |  |  |  |  |  |  |
| **Outer** | **Hill Reef** | 36.37 | 202.62 | 612.43 | 17.41 | 60.05 |

**Table S3** Comparison of GLMMs used to examine the differences in herbivorous fish abundance, biomass and biomass growth. Models are compared using the corrected Akaike Criterion (AICc). Shown are degrees of freedom (df), model maximum log-likelihood (logLik), AICc, change in AICc (Δ) and AICc weight (wAICc).

| **Response variable** | **Variables** | **df** | **logLik** | **AICc** | **Δ** | **wAICc** |
| --- | --- | --- | --- | --- | --- | --- |
|  |  |  |  |  |  |  |
| **Abundance (100m2)** | Shelf × Habitat  Shelf + Habitat  Shelf  Habitat  Null | 10  7  6  4  3 | -555.76  -561.63  -565.52  -603.47  -605.27 | 1133.27  1138.14  1143.68  1215.24  1216.73 | 0.00  4.87  10.41  81.98  83.46 | 0.91  0.08  0.01  0.00  0.00 |
|  |  |  |  |  |  |  |
| **Biomass (100m2)** | Shelf + Habitat  Habitat  Shelf × Habitat  Shelf  Null | 7  6  10  4  3 | -1136.13  -1137.88  -1133.73  -1171.95  -1174.24 | 2287.13  2288.41  2289.21  2352.20  2354.66 | 0.00  1.27  2.07  65.06  67.33 | 0.53  0.28  0.19  0.00  0.00 |
|  |  |  |  |  |  |  |
| **Biomass Growth (100m2)** | Shelf + Habitat  Habitat  Shelf × Habitat  Shelf  Null | 7  6  10  4  3 | -274.44  -276.72  -272.63  -320.45  -322.12 | 563.74  566.09  567.00  649.20  650.43 | 0.00  2.35  3.26  85.46  86.69 | 0.66  0.21  0.13  0.00  0.00 |
|  |  |  |  |  |  |  |
| **Productivity Consumed (100m2)** | Shelf + Habitat  Habitat  Shelf × Habitat  Shelf  Null | 7  6  10  4  3 | -682.11  -684.10  -681.00  -707.52  -710.39 | 1379.09  1380.86  1383.74  1423.35  1426.96 | 0.00  1.77  4.66  44.26  47.87 | 0.66  0.27  0.06  0.00  0.00 |
|  |  |  |  |  |  |  |
| **Standardized Abundance** | Shelf + Habitat  Shelf × Habitat  Habitat  Null  Shelf | 7  10  6  3  4 | -348.35  -345.26  -350.94  -444.84  -444.02 | 711.57  712.28  714.53  895.85  896.35 | 0.00  0.71  2.96  184.29  184.78 | 0.52  0.36  0.12  0.00  0.00 |
|  |  |  |  |  |  |  |
| **Standardized Biomass** | Shelf + Habitat  Habitat  Shelf × Habitat  Shelf  Null | 7  6  10  4  3 | -902.20  -903.56  -901.58  -964.06  -965.27 | 1819.27  1819.76  1824.90  1936.43  1936.73 | 0.00  0.49  5.63  117.16  117.46 | 0.54  0.42  0.03  0.00  0.00 |
| **Standardized Biomass Growth** | Shelf + Habitat  Habitat  Shelf × Habitat  Shelf  Null | 7  6  10  4  3 | -40.64  -42.47  -40.47  -150.48  -149.57 | 96.15  97.59  102.70  307.13  307.44 | 0.00  1.44  6.54  210.98  211.29 | 0.66  0.32  0.02  0.00  0.00 |
|  |  |  |  |  |  |  |
| **Standardized Productivity Consumed** | Shelf + Habitat  Habitat  Shelf × Habitat  Shelf  Null | 7  6  10  4  3 | -449.14  -450.75  -448.84  -485.84  -487.23 | 913.15  914.15  919.43  979.98  980.64 | 0.00  1.01  6.29  66.83  67.49 | 0.61  0.37  0.03  0.00  0.00 |


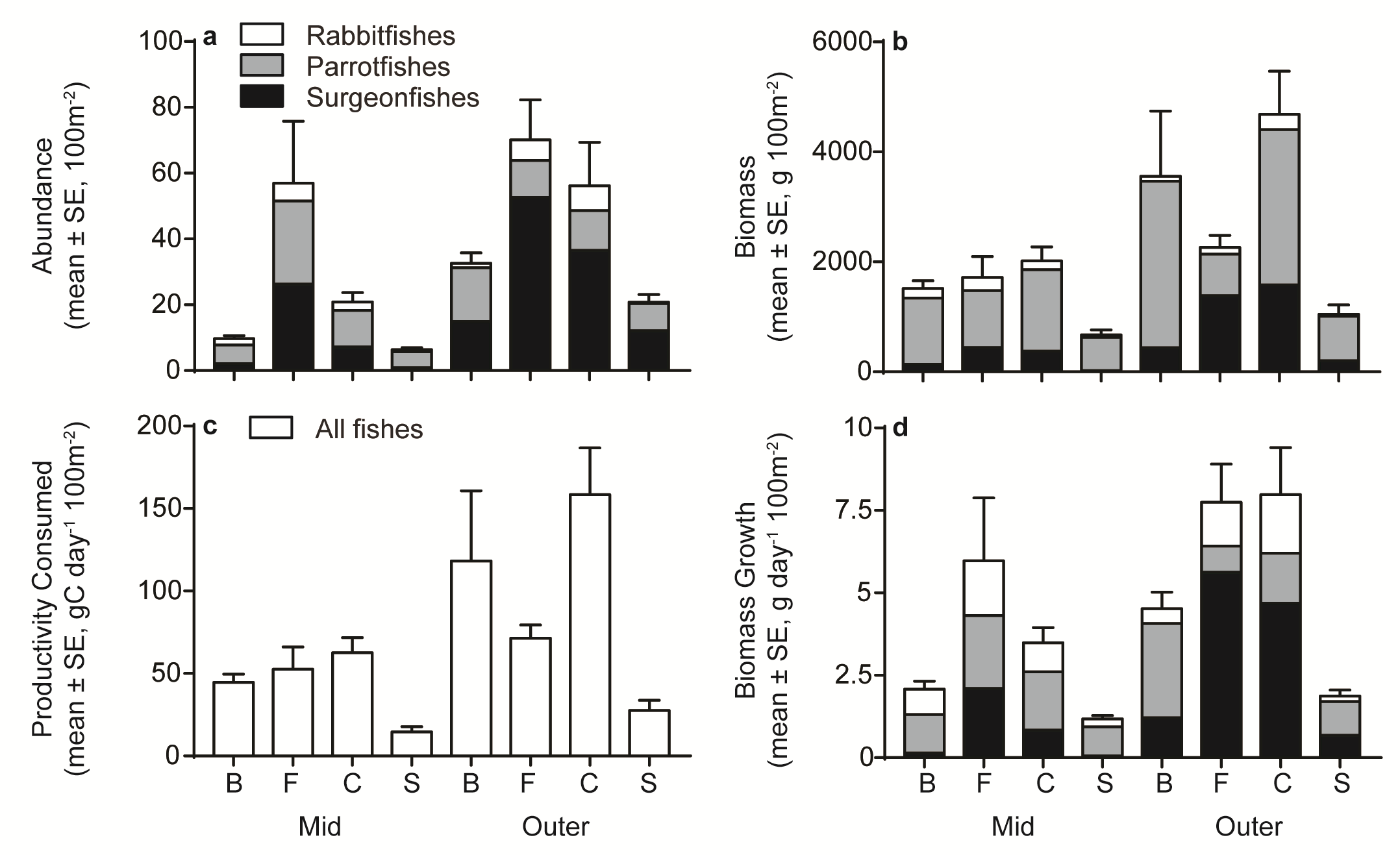


**Fig. S1** The mean (**a**) herbivorous fish abundance, (**b**) herbivorous fish biomass, (**c**) algal turf productivity consumed by herbivorous fishes and (**d**) herbivorous fish biomass growth, per 100 m2, across four reef habitats and two shelf positions on the Great Barrier Reef. **B** back reef, **F** reef flat, **C** reef crest, **S** reef slope.

**Table S4** Summary of generalized linear mixed effects model (GLMM) results used to examine the differences in fish abundance, biomass, biomass growth and productivity consumed. Models contain the fixed effects: shelf position (mid and outer) and habitat (slope, crest, flat, back). SE = standard error, negative binomial models use a *z* value while models with a Gamma distribution use a *t* value.

| **Response variable** | **Model used** | **Predictor variable** | **Estimate** | **SE** | ***z/t* value** | ***p* value** |
| --- | --- | --- | --- | --- | --- | --- |
|  |  |  |  |  |  |  |
| **Abundance (100m2)** | Negative Binomial (GLMM) | Intercept  Outer-Shelf  Back  Crest  Slope  Outer:Back  Outer:Crest  Outer:Slope | 3.8601  0.3312  -1.6245  -0.8395  -2.0417  0.9702  0.5231  0.8783 | 0.2213  0.3096  0.2263  0.2175  0.2306  0.3080  0.2994  0.3073 | 17.447  1.070  -7.180  -3.860  -8.855  3.150  1.747  2.858 | < 0.001  0.2847  < 0.001  < 0.001  < 0.001  < 0.01  0.0806  < 0.01 |
|  |  |  |  |  |  |  |
| **Biomass (100m2)** | Gamma  (GLMM) | Intercept  Outer-shelf  Back  Crest  Slope | 7.2795  0.5705  0.1045  0.4835  -0.8750 | 0.2092  0.2682  0.1480  0.1455  0.1429 | 34.79  2.13  0.71  3.32  -6.12 | < 0.001  < 0.05  0.480  < 0.001  < 0.001 |
|  |  |  |  |  |  |  |
| **Biomass Growth (100m2)** | Gamma  (GLMM) | Intercept  Outer-shelf  Back  Crest  Slope | 1.5551  0.5747  -0.7192  -0.2068  -1.4591 | 0.1804  0.2232  0.1424  0.1400  0.1379 | 8.621  2.574  -5.052  -1.477  -10.583 | < 0.001  < 0.05  < 0.001  0.14  < 0.001 |
|  |  |  |  |  |  |  |
| **Productivity Consumed (100m2)** | Gamma  (GLMM) | Intercept  Outer-shelf  Back  Crest  Slope | 3.7581  0.6689  0.0971  0.5302  -1.1822 | 0.2477  0.2934  0.2264  0.2226  0.2197 | 15.171  2.280  0.429  2.382  -5.381 | < 0.001  < 0.05  0.668  < 0.05  < 0.001 |
|  |  |  |  |  |  |  |
| **Standardized Abundance** | Negative binomial (GLMM) | Intercept  Outer-shelf  Back  Crest  Slope | 2.9454  0.8116  -2.5605  -2.6554  -2.1208 | 0.2305  0.3043  0.1860  0.1834  0.1668 | 12.781  2.667  -13.766  -14.478  -12.719 | < 0.001  < 0.01  < 0.001  < 0.001  < 0.001 |
|  |  |  |  |  |  |  |
| **Standardized Biomass** | Gamma (GLMM) | Intercept  Outer-shelf  Back  Crest  Slope | 6.6704  0.4802  -1.3460  -1.6108  -1.4604 | 0.2070  0.2639  0.1459  0.1431  0.1411 | 32.22  1.82  -9.23  -11.25  -10.35 | < 0.001  < 0.069  < 0.001  < 0.001  < 0.001 |
|  |  |  |  |  |  |  |
| **Standardized Biomass Growth** | Gamma (GLMM) | Intercept  Outer-shelf  Back  Crest  Slope | 0.9518  0.4823  -2.1787  -2.3032  -2.0488 | 0.1771  0.2173  0.1402  0.1380  0.1360 | 5.373  2.220  -15.539  -16.692  -15.063 | < 0.001  < 0.05  < 0.001  < 0.001  < 0.001 |
|  |  |  |  |  |  |  |
| **Standardized Productivity Consumed** | Gamma  (GLMM) | Intercept  Outer-shelf  Back  Crest  Slope | 3.1467  0.5784  -1.3508  -1.5614  -1.7633 | 0.2467  0.2893  0.2247  0.2203  0.2183 | 12.755  2.000  -6.012  -7.087  -8.078 | < 0.001  < 0.05  < 0.001  < 0.001  < 0.001 |


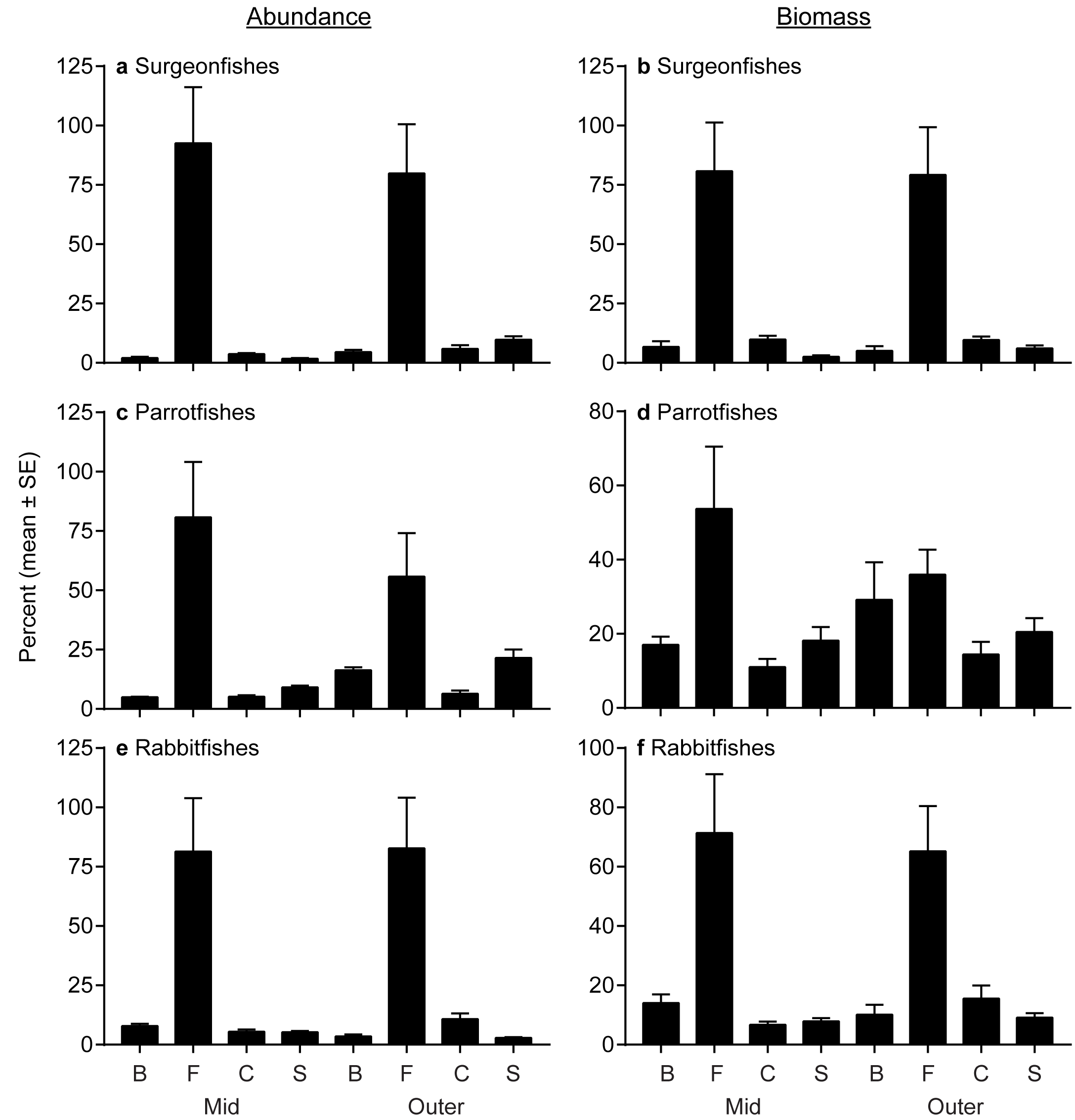


**Fig. S2** The mean percentage of surgeonfish (**a**) abundance and (**b**) biomass, parrotfish (**c**) abundance and (**d**) biomass, and rabbitfish (**e**) abundance and (**f**) biomass in four reef habitats across two shelf positions on the Great Barrier Reef. **B** back reef, **F** reef flat, **C** reef crest, **S** reef slope.

**Text S2** Relationship between fish abundance/biomass and water movement.

We also considered the relationship between grazing fish abundance or biomass with water movement (net current velocity) for the three focal groups (surgeonfishes, parrotfishes and rabbitfishes). Mean values of net current velocity in each reef habitat on each shelf position were obtained from the literature (Fulton & Bellwood 2005). Where values were not available for a particular habitat they were approximated based on the differences in water flow velocity experienced on mid- vs. outer-shelf reef crests (Fulton et al. 2013). The relationships between the mean abundance and biomass of each grazing fish group and mean net current velocity were examined using linear regression.

A strong correlation exists between wave action (net current velocity) and abundance or biomass of grazing herbivores (Fig. S2). This is most clearly seen in the surgeonfishes (R2 = 0.69, *p* <0.05 for abundance; R2 = 0.8, *p* <0.01 for biomass Table S5; Fig. S2a, b). Scraping parrotfishes and rabbitfishes also exhibit positive relationships, although they are relatively weaker with only the rabbitfishes exhibiting a significant relationship between abundance and current velocity (R2 = 0.78, *p* <0.01; Table S5; Fig. S2c, d, e, f).

**References**

Fulton, C.J. & Bellwood, D.R. (2005) Wave-induced water motion and the functional implications for coral reef fish assemblages. *Limnology and Oceanography*, **50**, 255–264.

Fulton, C.J., Binning, S.A., Wainwright, P.C., Bellwood, D.R., (2013) Wave-induced abiotic stress shapes phenotypic diversity in a coral reef fish across a geographical cline. *Coral Reefs*, **32**, 685–689.

**Table S5** Summary of linear regression results used to examine the relationship between fish abundance and biomass with water movement (net current velocity). SE = standard error.

| **Response variable** | **Predictor variable** | **R2** | **Estimate** | **SE** | ***F* value** | ***p* value** |
| --- | --- | --- | --- | --- | --- | --- |
|  |  |  |  |  |  |  |
| **Surgeonfish abundance** | Current speed | 0.69 | 0.7816 | 0.216 | 13.09 | < 0.05 |
|  |  |  |  |  |  |  |
| **Parrotfish abundance** | Current speed | 0.08 | 0.0978 | 0.133 | 0.5411 | 0.4897 |
|  |  |  |  |  |  |  |
| **Rabbitfish abundance** | Current speed | 0.78 | 0.1266 | 0.0272 | 21.66 | < 0.01 |
|  |  |  |  |  |  |  |
| **Surgeonfish biomass** | Current speed | 0.82 | 27.53 | 5.204 | 27.99 | < 0.01 |
|  |  |  |  |  |  |  |
| **Parrotfish biomass** | Current speed | 0.04 | 10.36 | 19.56 | 0.2806 | 0.6153 |
|  |  |  |  |  |  |  |
| **Rabbitfish biomass** | Current speed | 0.32 | 2.555 | 1.506 | 2.88 | 0.1406 |

­


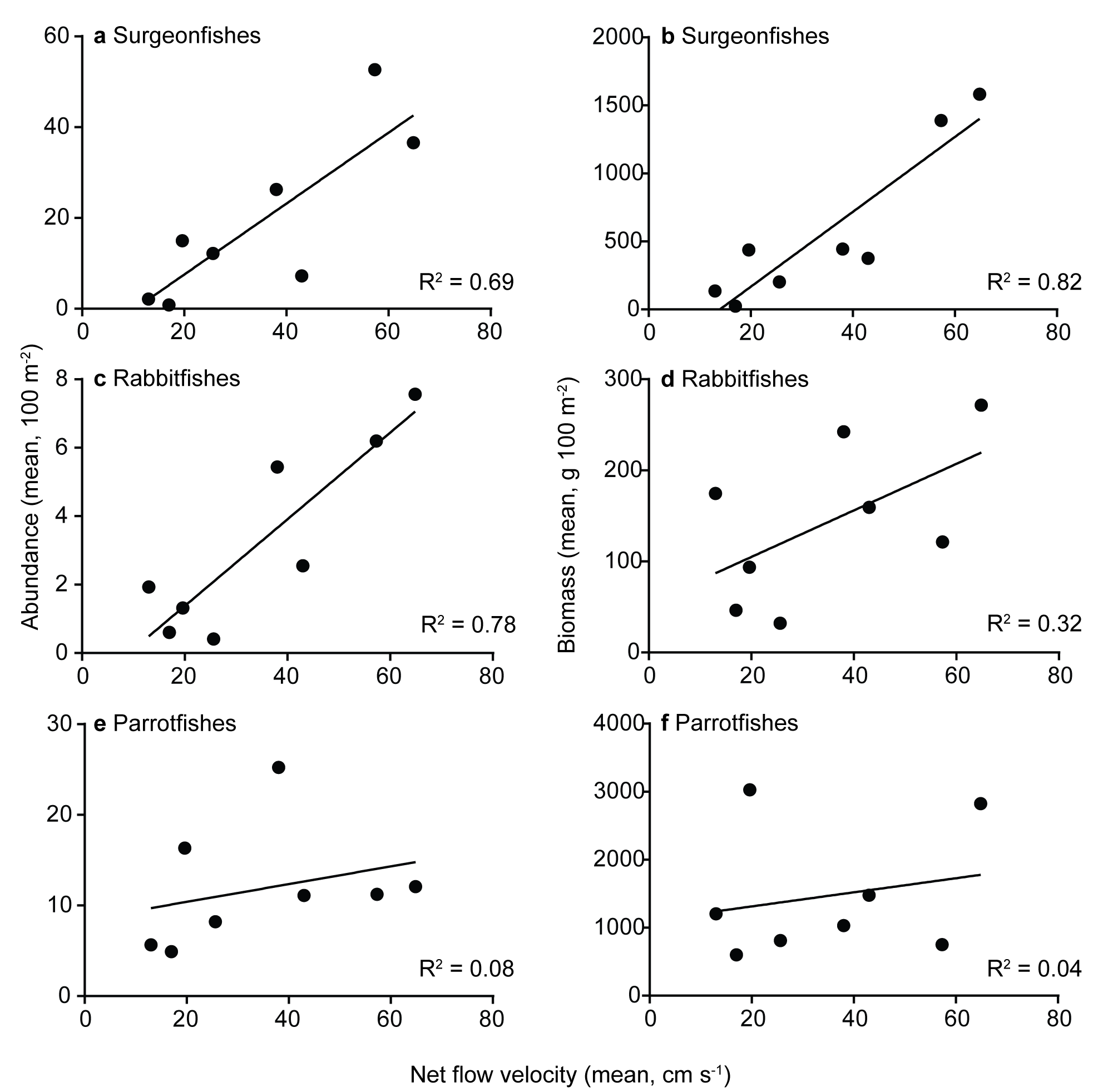


**Fig. S3** The relationship between surgeonfish (**a**) abundance, (**b**) biomass, rabbitfish (**c**) abundance, (**d**) biomass and parrotfish (**e**) abundance, (**f**) biomass and water movement (mean net current velocity).

**Text S3** Estimating grazing fish population increases.

To estimate how much grazing fish populations could have increased once grazing fishes entered shallow water coral reef environments, we divided the percentage of the grazing fish population on mid-shelf reef shallow water habitats (the crest and flat) by the percentage of the population on the deeper water habitats (the slope and back reef) i.e. 90.4% divided by 9.6%. This suggested that population sizes may have increased by approximately 940%.
